# CD19 Is Internalized Together with IgM in Proportion to B Cell Receptor Stimulation and Is Modulated by Phosphatidylinositol 3-Kinase in Bone Marrow Immature B Cells

**DOI:** 10.4049/immunohorizons.2200092

**Published:** 2023-01-01

**Authors:** Megan R. McCaleb, Anjelica M. Miranda, Kaysie C. Ratliff, Raul M. Torres, Roberta Pelanda

**Affiliations:** * Department of Immunology and Microbiology, University of Colorado School of Medicine, Anschutz Medical Campus, Aurora, CO;; † Department of Immunology and Genomic Medicine, National Jewish Health, Denver, CO

## Abstract

Newly generated immature B cells that bind self-antigen with high avidity arrest in differentiation and undergo central tolerance via receptor editing and clonal deletion. These autoreactive immature B cells also express low surface levels of the coreceptor CD19, a key activator of the PI3K pathway. Signals emanating from both CD19 and PI3K are known to be critical for attenuating receptor editing and selecting immature B cells into the periphery. However, the mechanisms that modulate CD19 expression at this stage of B cell development have not yet been resolved. Using in vivo and in vitro models, we demonstrate that *Cd19* de novo gene transcription and translation do not significantly contribute to the differences in CD19 surface expression in mouse autoreactive and nonautoreactive immature B cells. Instead, CD19 downregulation is induced by BCR stimulation in proportion to BCR engagement, and the remaining surface IgM and CD19 molecules promote intracellular PI3K-AKT activity in proportion to their level of expression. The internalized CD19 is degraded with IgM by the lysosome, but inhibiting lysosome-mediated protein degradation only slightly improves surface CD19. In fact, CD19 is restored only upon Ag removal. Our data also reveal that the PI3K-AKT pathway positively modulates CD19 surface expression in immature B cells via a mechanism that is independent of inhibition of FOXO1 and its role on *Cd19* gene transcription while is dependent on mTORC1. *ImmunoHorizons*, 2023, 7: 49–15.

## INTRODUCTION

Central B cell tolerance is the biological mechanism that prevents newly generated B cells with high avidity for common self-antigens from exiting the bone marrow, thereby reducing the autoreactive capacity of the circulatory B cell repertoire and the possibility of developing autoimmunity. In both mice and humans, 55–75% of the newly generated IgM^+^ immature B cells in the bone marrow are autoreactive (1, 2). About half of these cells express high-avidity BCRs and are regulated by central tolerance via receptor editing and clonal deletion (reviewed in Refs. 3–5). During this central selection checkpoint, only immature B cells that do not bind self-antigen or that bind self-antigen with low avidity exit the bone marrow and continue their differentiation. The egress of immature B cells from the bone marrow and their differentiation into transitional and mature B cells are dependent on a tonic signal that is generated by a signaling-competent BCR independent of ligand or following low binding to a ligand (6–10). Self-reactive immature B cells chronically binding self-antigen display reduced tonic signaling as a result of Ag-induced internalization of the BCR (11). The absence of tonic BCR signaling, in addition to self-antigen–induced BCR signaling, prevents self-reactive B cells from terminating receptor editing and entering the circulation (8, 9, 11).

Our group has used the 3–83Igi mouse model of central B cell tolerance to discover molecular pathways that are more active in nonautoreactive (i.e., naive) than self-reactive immature B cells and that mediate their differential selection (9, 12–16). In 3–83Igi,H-2^b^ mice in which B cells develop in the chronic presence of the K^b^ self-antigen, immature B cells substantially internalize the IgM BCR, arrest in differentiation, express high levels of DNA recombinases Rag1 and Rag2, and undergo receptor editing (12, 14, 17). During receptor editing, additional Ig VJ gene recombination events at the L chain loci exchange the autoreactive L chain and produce a new BCR with no (or weak) self-reactivity. The result of receptor editing is IgM^+^ edited B cells that are released into the circulation (12, 14). One of the biochemical pathways displaying higher activity in 3–83Igi B cells developing in the absence of self-antigen relative to when they develop in the presence of the K^b^ Ag is the lipid kinase PI3K, for which activity was detected as p-AKT (18). Importantly, PI3K activity has meaningful functional consequences in central B cell selection. Indeed, the expression of a synthetic constitutively active form of PI3Kα (P110*) in B cells of 3–83Igi,H-2^b^ mice completely breached central tolerance, allowing high-avidity 3–83 autoreactive B cells to exit the bone marrow and enter the periphery, where they underwent further maturation (18). These findings were generally recapitulated by the deletion of the PI3K inhibitor PTEN (19), a protein that is upregulated in autoreactive 3–83 cells (5). Conversely, it has also been shown that deletion of PI3K provokes receptor editing even in the absence of BCR stimulation (20, 21). Thus, examining how PI3K is regulated in immature B cells is essential for understanding how central B cell tolerance is either maintained or breached.

The BCR signaling cascade activates PI3K-AKT via CD19-dependent and CD19-independent mechanisms, although available data suggest that the CD19-dependent mechanism plays a more dominant role in AKT activation (22–26). Upon BCR stimulation, surface CD19 moves in closer proximity to IgM (27, 28) and becomes tyrosine phosphorylated by Lyn and/or other Src family kinases (23, 29, 30). Once phosphorylated, CD19 recruits multiple signaling molecules to amplify BCR signal transduction (31, 32), and phosphorylation of CD19 Y482 and Y513 is required for the recruitment and activation of PI3K (25). Once PI3K is activated, this leads to the production of the phospholipid phosphatidylinositol-(3,4,5)-trisphosphate, which coordinates at the plasma membrane the activation of many pathways, including that of protein kinase B, or AKT (33–35). Likely due to its role in positively regulating PI3K signaling, CD19 defects have important consequences in B cells, including during their bone marrow development (reviewed in Ref. 36). In fact, deletion of CD19 during B cell development leads to a significant decrease in B cell maturation and bone marrow egress, whereas it increases *Rag1/2* gene expression and receptor editing (36–40).

It has been previously reported that 3–83 immature B cells developing in the presence of self-antigen express much lower levels of surface CD19 than in the absence of cognate Ag (17, 20). This observation extends to self-reactive human immature B cells developing in human immune system (humanized) mice (41). Based on the above review of the literature on CD19 and PI3K, these observations suggest that self-reactive immature B cells may downmodulate CD19 as a mechanism to reduce PI3K activity and undergo receptor editing. Despite the significance of these observations, the mechanisms that control and modulate CD19 expression in immature B cells have not yet been elucidated.

In this study, we investigated potential ways by which immature B cells modulate CD19. We show that CD19 downregulation is independent of transcriptional and posttranscriptional mechanisms, whereas it is dependent on strong BCR cross-linking that leads to both BCR and CD19 internalization and their lysosomal degradation. Furthermore, we discovered that the recovery of CD19 on the cell surface is contingent on the complete removal of BCR stimulation, but is also positively modulated by the PI3K-AKT-mTORC1 pathway.

## MATERIALS AND METHODS

### Mice

Ig knock-in 3–83Igi,H-2^d^ and 3–83Igi,H-2^b^ mice on a BALB/c genetic background have been previously described (9, 13–15). In 3–83Igi mice, all developing B cells harbor prerearranged 3–83 Ig H and κ L chain genes and express a BCR with high avidity for the Ag MHC-I H-2 K^b^ (42, 43); in these mice, >80% of bone marrow B220^+^IgD^−^ B cells are immature B cells expressing the 3–83 IgM BCR (13, 18), which is autoreactive in the H-2^b^ genetic background and nonautoreactive in the H-2^d^ genetic background. C57BL/6 and CB17,H-2^b^ mice (BALB/c genetic background congenic for H-2^b^; generated in-house) were used as wild-type controls. The 3–83Igi P110*-mb1Cre H-2^d^ and H-2^b^ (BALB/c) mice and CD19 knockout (BL/6, CD19^cre/cre^) mice have been described previously (18, 44). FOXO1^fl/fl^ mice and Rosa26-FOXO1^AAA^ (BL/6) mice, which were received from Dr. Stephen Hedrick (University of California, San Diego) and previously described (45, 46), were crossed to 3–83Igi,H-2^b^-mb1-Cre mice and 3–83Igi,H-2^d^-mb1Cre mice, respectively, to generate autoreactive 3–83Igi,H-2^b^-FOXO1^fl/fl^-mb1Cre mice and nonautoreactive 3–83Igi,H-2^d^-Rosa26-FOXO1^AAA^-mb1Cre mice. Bone marrow cells from MD4, MD4 × ML5, and Ars/A1 (BL/6) mice were a gift from Dr. Andy Getahun (University of Colorado Anschutz Medical Campus, Aurora, CO) and have been previously described (47, 48). All mice were bred and maintained in a specific pathogen-free facility at the University of Colorado Anschutz Medical Campus Vivarium and used for experiments (both females and males in approximately equal numbers) between 6 and 20 wk of age. All animal procedures were approved by the University of Colorado Denver Institutional Animal Care and Use Committee.

### Bone marrow B cell culture and treatment

Bone marrow cells were extracted from femurs and pelvises of euthanized mice, and single-cell suspensions were incubated for 3 min in ACK lysis buffer (0.15 M NH_4_Cl, 0.01 M KHCO_3_, and 0.1 mM EDTA [pH 7.2–7.4]) to remove erythrocytes. B cells were enriched by positive selection using anti-B220 mAbs coupled to magnetic beads (Miltenyi Biotec) and an AutoMACS (Miltenyi Biotec) according to the manufacturer’s instructions. B cell purity was consistently >95% based on B220 staining. Enriched B cells (or total bone marrow cells for experiments with ERK, AKT, and mTORC1 inhibitors) were cultured at 2 × 10^6^ cells/ml at 37°C, 5% CO_2_, in complete IMDM (5% FBS, 1% GlutaMAX, 1% penicillin-streptomycin, 1% nonessential amino acids, 0.1 M 2-ME) for times indicated in the figure legends. The following inhibitors were added to the bone marrow B cell cultures: actinomycin D (0.1 μM; Sigma-Aldrich), afuresertib (5 μM; Sigma-Aldrich), bafilomycin (12 nM; Sigma-Aldrich), bortezomib (0.5 μM; Selleck Chemicals), cycloheximide (2 μM; Sigma-Aldrich), FR180204 (30 μM; Santa Cruz Biotechnology), and rapamycin (10 μM; EMD Millipore). The following Abs were added for B cell stimulation: anti-3–83Ig idiotypic Ab S23 (49) (produced in-house) at ≤10 μg/ml (as indicated in figures) and anti-IgM F(ab’)_2_ Abs (SouthernBiotech) at 5 μg/ml. DMSO and/or PBS diluents were added in control cultures.

### Bone marrow B cell culture on stromal cell layer

To prepare stromal cells, total bone marrow cells from H-2^d^ or H-2^b^ mice were isolated and depleted of erythrocytes with ACK lysis buffer, as described in the *Materials and Methods*. Bone marrow stromal cell layers were prepared by plating 4 × 10^6^ bone marrow cells/well in a 24-well tissue culture plate in complete IMDM. The plate was incubated at 37°C for 2 d to allow stromal cells to sufficiently adhere to the plate and other cells to die off. The media was exchanged every 2 to 3 d to remove dead cells and add fresh nutrients. Once stromal cells were at 90% confluency (around day 8), the media above the stromal cells was vacuumed out, and 500 μl of media was added to each well carefully to not disrupt the stromal cell layer. Bone marrow B cells from 3–83Igi H-2^d^ or H-2^b^ mice were enriched by B220-positive magnetic cell selection (Miltenyi Biotec) and added at 1.5 × 10^6^ cells/0.5 ml/well on top of the bone marrow stromal cell layer for a total of 1 ml media/well. B cells on stromal cell layers were incubated at 37°C, 5% CO_2_, for the time points indicated in the figure legends. B cells were removed from the stromal layer for analysis or transferred into a new well by carefully pipetting up and down the floating cells with minimal disturbing of the adherent stromal cells.

### Quantitative real-time PCR

Ex vivo bone marrow B cells were purified either as B220^+^ cells by positive selection using anti-B220 Abs coupled to magnetic beads or as B220^+^IgD^−^ cells first by negative selection with anti-IgD Abs conjugated to PE fluorophore followed by anti-PE Abs coupled to magnetic beads and then by positive selection with anti-B220 Abs coupled to magnetic beads. Cell enrichment was performed with an autoMACS (Miltenyi Biotec) according to the manufacturer’s instructions. B cell purity was consistently >95% based on B220 and IgD staining. Total RNA was purified using the RNeasy Plus Mini Kit (Qiagen), and cDNA was synthesized using the SuperScript IV VILO Master Mix (Thermo Fisher Scientific). Murine *Cd19* (Mm001278670_g1), *Cd81* (Mm00504870_m1), *Rag1* (Mm01270936_m1), *Rag2* (Mm00501300_m1), and *Foxo1* (Mm00490671_m1) genes were amplified from cDNAs using TaqMan primer (Applied Biosystems) and probe sets purchased from Thermo Fisher Scientific. Differences in specific mRNA levels were determined by RT-PCR using the comparative threshold cycle (ΔΔCt), and each sample was normalized to murine *Cd79b* (Mm00434143_m1, TaqMan; Applied Biosystems). All samples were run in duplicate using the QuantStudio 7 Flex Real-Time PCR System (Thermo Fisher Scientific).

### Flow cytometry

Bone marrow single-cell suspensions depleted of erythrocytes with ACK lysis buffer were stained with fluorochrome or biotinconjugated Abs against mouse B220 (RA3–6B2), IgD (11-26c-2a), IgM (eB121-15F9), CD2 (RM2-5), CD21 (7E9), CD23 (B3B4), CD24 (M1/69), CD19 (1D3), CD81 (Eat2), and Igκ (H139–52.1) purchased from eBioscience, BD Pharmingen, BioLegend, or R&D Systems. Biotin-labeled Abs were visualized with fluoro-chrome-conjugated streptavidin (BD Biosciences). The Ghost Dye 780 (eBioscience) was used to discriminate unfixed dead cells. Staining for intracellular CD19 and total (intracellular plus surface) CD19, Igκ, and IgM was performed on cells after they were stained for surface markers and after treatment with BioLegend Fixation Buffer and BD Perm/Wash buffer. Surface and intracellular CD19 pools were distinguished by first staining surface CD19 and then, after fixation and permeabilization, by staining intracellular CD19 with the same anti-CD19 mAb clone in different colors. Staining with Abs to p-AKT-T308 (C31E5E) and FOXO1 (C29H4) (Cell Signaling Technology) and with BD Biosciences Ab to PTEN (A2B1) was performed on cells fixed with the BioLegend Fixation Buffer and permeabilized in 90% methanol. When staining for p-AKT, all staining Abs were used after cell fixation and permeabilization to avoid cell signaling. The Zombie UV Fixable Viability Kit from BioLegend was used to discriminate dead cells in fixed and methanol-permeabilized samples. GFP fluorescence marked the expression of FOXO1-AAA and P110* proteins in Cre-expressing FOXO1-AAA and P110* mice, respectively. Data acquisition was done on the Aurora cytometer (Cytek Biosciences) and analyzed with FlowJo software (BD Biosciences). Analyses were performed on live cells based on the incorporation of Ghost Dye 780, Zombie UV, or forward and side scatter. B cells were identified by the expression of the pan B cell marker B220. Cell doublets were excluded based on the forward scatter area and forward scatter height for data analyzed on the Aurora cytometer.

### Statistical data analysis

Data were analyzed using Prism software (GraphPad). Statistical significance for normally distributed data was determined by a one-tailed paired *t* test if data were from the same mouse and a one-tailed unpaired *t* test if data were from different mice. For the unpaired *t* test, Welch correction was added for groups that had unequal variance. Data that were not normally distributed were analyzed with the nonparametric Mann-Whitney *U* test if unpaired or the Wilcoxon test if paired. The one-sample *t* test was used for experiments with high technical variation that analyzed one mouse per group per experiment. In these analyses, data were normalized against one control mouse in each experiment and then analyzed by one-sample *t* test against a theoretical value of 1. Simple linear regression analyses were applied to measure correlation in the expression of markers. Data in bar and line graphs are represented as means 6 SD. Significance levels are labeled as *p ≤ 0.05, **p ≤ 0.01, ***p ≤ 0.001, and ****p ≤0.0001; *p* > 0.05 was not significant. All experimental replicates are biological, not technical. Investigators were not blinded to samples.

## RESULTS

### Autoreactive immature B cells downregulate CD19 independent of changes in *CD19* gene transcription and alternative splicing

**FIGURE 1.**
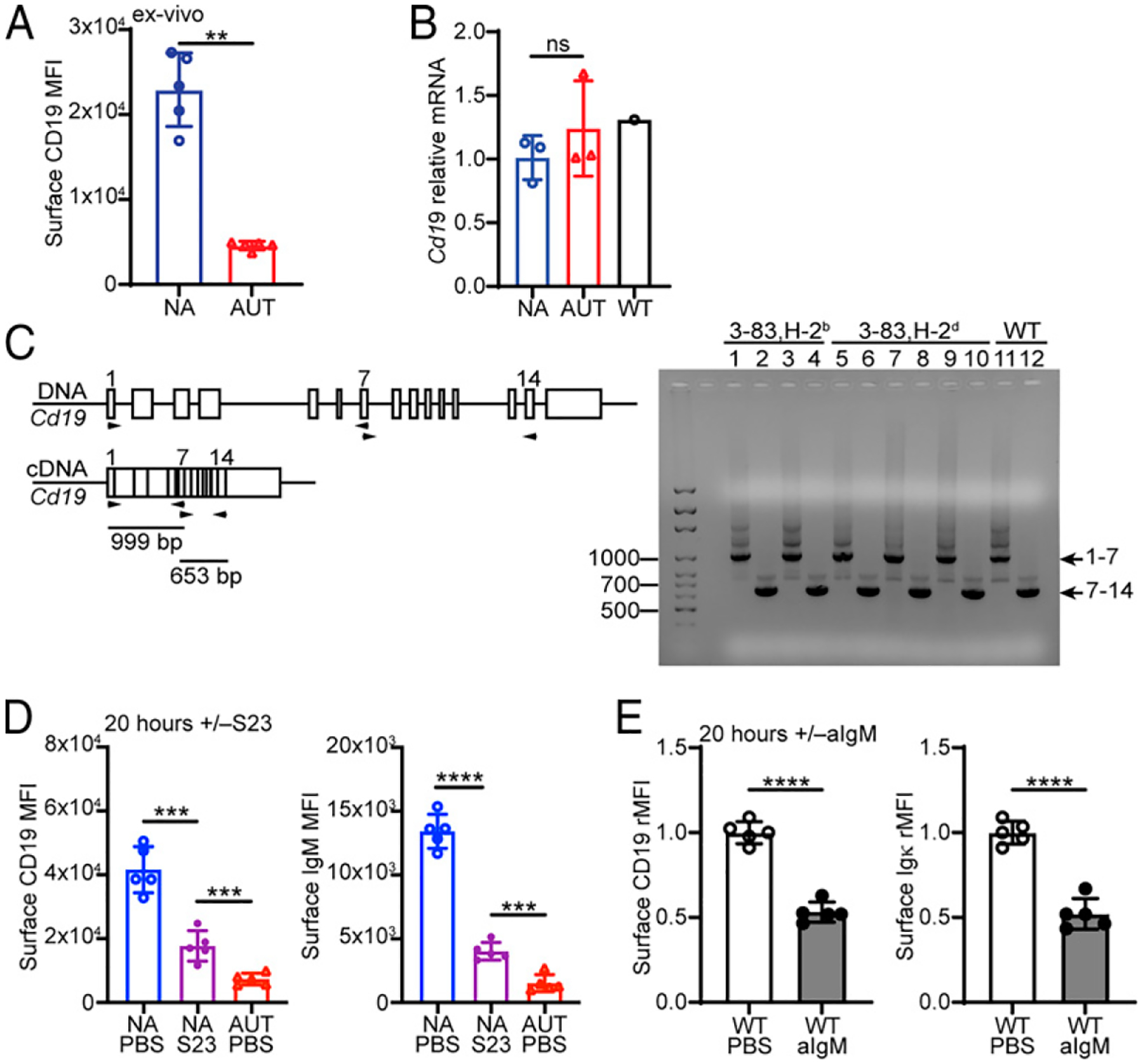
Surface CD19 is downmodulated in BCR-stimulated immature B cells independently of *Cd19* gene transcription and splicing. (**A**) Mean 6 SD of surface CD19 fluorescence intensity (MFI) on B220^+^CD24^high^CD23^−^ bone marrow immature B cells from 3–83Igi,H-2^b^ autoreactive (AUT; red) and 3–83Igi,H-2^d^ nonautoreactive (NA; blue) mice (gating strategy of immature B cells is shown in Supplemental Fig. 1A). *n* = 5 mice/group in five independent experiments; *p* values were calculated by Mann–Whitney *U* test. (**B**) Relative *Cd19* mRNA levels (mean 6 SD) in B220^+^IgD^−^ bone marrow cells of NA (*n* = 3) and AUT (*n* = 3) 3–83Igi mice and one WT CB17 mouse control. Cells were isolated from the mice in three independent experiments, and all samples were run by RT-PCR at the same time to minimize technical variations. *Cd19* mRNA was normalized to *Cd79b* mRNA in each sample, and the normalized *Cd19* mRNA levels are expressed as fold change over the average *Cd19* mRNA levels in NA cells. The *p* values were calculated using a Mann–Whitney *U* test. (**C**) Left, Schematic of the mouse *Cd19* genomic and mRNA structures (in scale), indicating the location of the primers used to amplify exons 1–7 and 7–14. (C) Right, Agarose gel analysis of *Cd19* mRNA exons 1–7 and 7–14 amplified on cDNA from B220^+^IgD^−^ bone marrow cells of AUT (H-2^b^; *n* 5 = 2) and NA (H-2^d^; *n* = 3) 3–83Igi mice and one WT control mouse. (**D**) Mean MFI 6 SD of surface CD19 and IgM of immature B cells from 3–83Igi,H-2^d^ NA mice (*n* = 5) after 20 h of culture with or without (i.e., PBS) 10 mg/ml of anti-3–83Ig S23 antibodies and relative to immature B cells from 3–83Igi,H-2^b^ AUT mice (*n* = 5). Data are from five experiments, and *p* values were calculated using a paired *t* test for the NA mice and an unpaired *t* test for AUT mice. (**E**) Relative surface CD19 and Igκ on B220^+^CD24^high^CD2^1^CD23^−^IgD^−^Igκ^+^ immature B cells from WT CB17 mice (*n* = 5, analyzed in four independent experiments) after 20 h of culture with 5 μg/ml of anti-IgM F(ab’)_2_ antibodies relative to control with PBS. Relative MFI (rMFI) data were calculated by dividing the MFI of cells treated with anti-IgM for each individual mouse by the average MFI of cells treated with PBS. The *p* values were calculated using a paired *t* test. In all bar graphs, **p ≤ 0.01, ***p ≤ 0.001, ****p ≤ 0.0001.

Flow cytometric analyses of CD19 indicated surface expression was ~5-fold lower on ex vivo 3–83 autoreactive (3-83Igi,H-2^b^ mice) bone marrow immature B cells (gated as B220^+^CD24^high^ CD23^−^) relative to nonautoreactive (3-83Igi,H-2^d^ mice) cells (Fig. 1A, gating strategy in Supplemental Fig. 1A). Real-time quantitative PCR analyses revealed no differences in the amount of *Cd19* transcripts between autoreactive and nonautoreactive 3–83 immature B cells that were magnetically enriched as B220^+^IgD^−^(Fig. 1B). Using primers that independently amplified *Cd19* exons 1–7 and 7–14 (Fig. 1C, left), we also found no differences in the size of *Cd19* transcripts between nonautoreactive and autoreactive B cells (Fig. 1C, right). Thus, the differences observed in CD19 protein expression do not appear to depend on differences in *Cd19* gene transcription or splicing.

To begin to explore the parameters by which BCR engagement leads to downregulation of CD19, we used the agonistic anti-3–83Ig idiotypic Ab S23 to promote BCR signaling in 3–83Igi,H-2^d^ (nonautoreactive) immature B cells in culture (49). Both CD19 and IgM were downmodulated from the cell surface of 3–83 immature B cells after 4 h (Supplemental Fig. 1B) or 20 h (Fig. 1D) of S23 stimulation, although not to the same extent observed on 3–83 autoreactive immature B cells that had been chronically exposed to K^b^ in the bone marrow of 3–83Igi,H-2^b^ mice (autoreactive PBS in Fig. 1D, Supplemental Fig. 1B). Importantly, CD19 downregulation was not limited to B cells from 3–83Igi mice, as wild-type bone marrow immature B cells treated with anti-IgM Abs displayed comparable downmodulation of CD19 (Fig. 1E).

CD19 associates and forms a complex with both CD81 and CD21, and this complex is required for CD19 surface expression in mature B cells (reviewed in Ref. 50). There was no difference in the transcription level of *Cd81* between autoreactive and nonautoreactive 3–83 immature B cells (Supplemental Fig. 1C). In addition, CD81 protein was expressed at very low levels in murine immature B cells when compared with mature B cells (Supplemental Fig. 1D) (41). Furthermore, mouse immature B cells express little to no CD21 (51), whether they are autoreactive or not (Supplemental Fig. 1E). Of note, bone marrow mature recirculating B cells, which express both CD81 and CD21, also exhibited downmodulation of CD19 and IgM after 20 h of Ab-mediated BCR stimulation (Supplemental Fig. 1F). Thus, downmodulation of CD19 from the surface of immature B cells following self-antigen or Ab-mediated BCR engagement is not related to changes in *Cd19* gene transcription or splicing or to the expression of molecules known to influence CD19 surface expression in mature B cells.

### Downregulation of CD19 increases with stronger BCR engagement and correlates with markers of receptor editing

**FIGURE 2.**
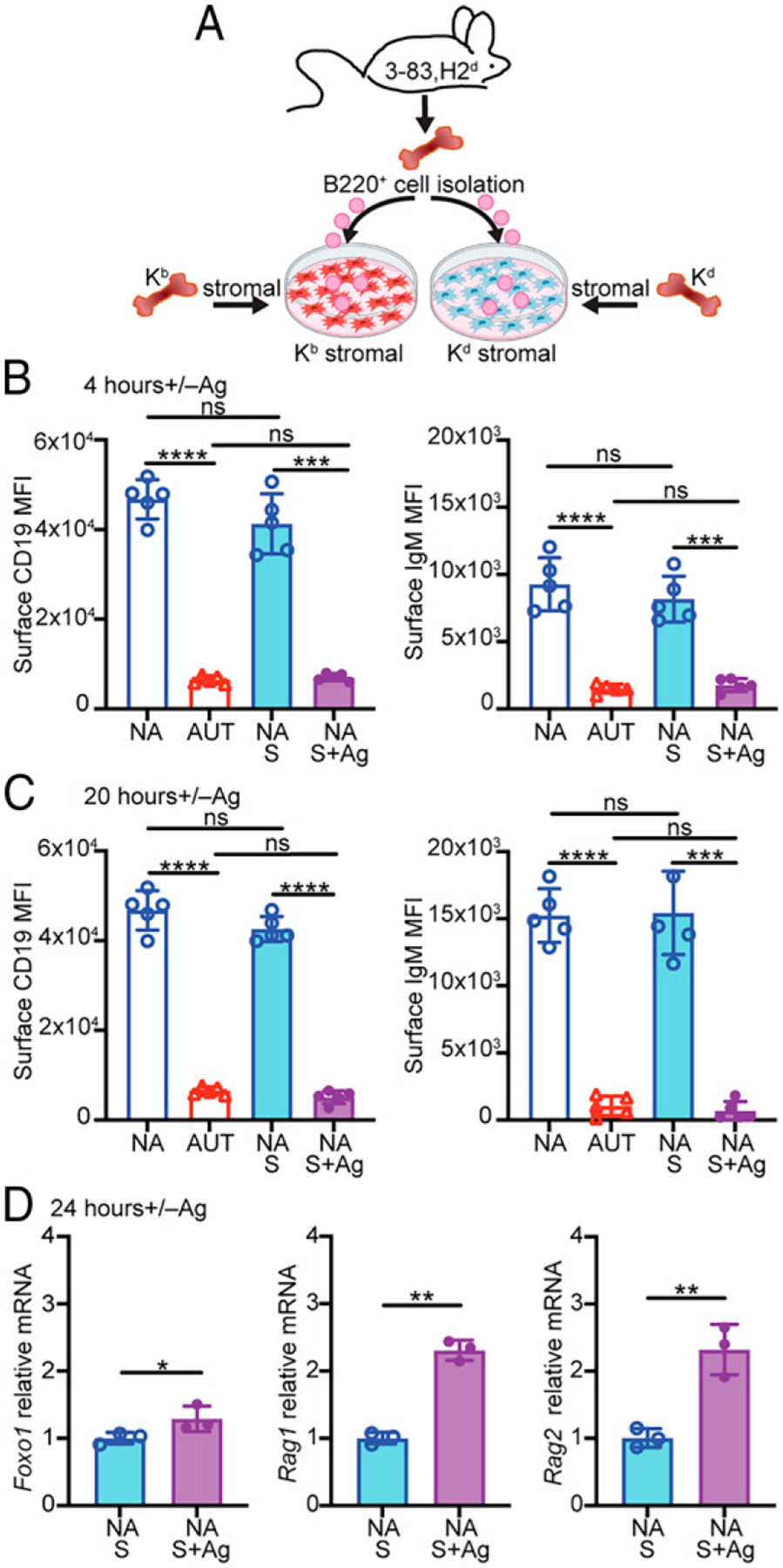
Stronger BCR stimulation induces larger CD19 downregulation and correlates with markers of receptor editing. (**A**) Schematic of stromal Ag stimulation of bone marrow B cells from 3–83Igi,H-2^d^ nonautoreactive (NA) mice. Mean MFI ± SD of surface CD19 and IgM on B220^+^CD24^high^CD23^−^ immature B cells (gated as in Supplemental Fig. 1A) from NA mice, cultured for 4 h (**B**) or 20 h (**C**) on stromal cell layers expressing either K^b^ (NA+S+Ag) or K^d^ (NA+S) and from ex vivo 3–83Igi autoreactive (AUT) and NA immature B cells (*n* = 5 mice/group in five independent experiments). (**D**) Relative *Foxo1*, *Rag1*, and *Rag2* mRNA levels (mean ± SD) in NA B220^+^ cells (*n* = 3 in two experiments) cultured for 24 h over K^b^ or K^d^ stromal cell layers. Normalized mRNA levels are expressed as fold change over the average mRNA levels of NA cells cultured on K^d^ stromal cell layer. The *p* values in all bar graphs were calculated using a paired *t* test for all comparisons, except when compared with AUT ex vivo cells in (C), in which an unpaired *t* test was used. In all bar graphs, *p ≤ 0.05, **p ≤ 0.01, ***p ≤ 0.001, ****p ≤ 0.0001.

To address whether the lower amount of CD19 downmodulation mediated by the S23 Ab in vitro was related to differences in the degree of BCR stimulation, we cultured bone marrow B cells from 3–83Igi,H-2^d^ mice on confluent layers of bone marrow stromal cells that expressed either the cognate Ag K^b^ (for a high-avidity interaction) or the nonreactive Ag K^d^ (Fig. 2A). Notably, after either 4 or 20 h of culture on a K^b^ stromal cell layer, surface CD19 and IgM were downregulated to the same extent measured on ex vivo 3–83,H-2^b^ autoreactive immature B cells (Fig. 2B, 2C). In addition, stimulation of 3–83 bone marrow B cells with a 3-fold serial dilution of S23 Ab caused detectable CD19 downmodulation starting at 0.7 ng/ml of S23 Ab and followed a dose-response thereafter (Supplemental Fig. 2A).

In developing bone marrow B cells, PI3K negatively regulates FOXO1 through AKT-mediated phosphorylation and protein degradation. This leads to reduced gene expression of *Foxo1* itself and of *Rag1* and *Rag2* (18, 20, 21, 24, 25, 52, 53). To determine whether CD19 downmodulation mediated by BCR stimulation correlates with changes in the expression of downstream targets of the PI3K-AKT-FOXO1 signaling pathway, we measured the transcript levels of *Foxo1*, *Rag1*, and *Rag2* in 3–83,H-2^d^ B cells cultured overnight on the K^b^ stromal cells. Indeed, Ag-mediated stimulation of 3–83 B cells led to significantly higher *Foxo1* and *Rag1/*2 transcription (Fig. 2D), suggesting reduced PI3K activity.

Overall, these data indicate that immature B cells downregulate CD19 in relation to the extent of BCR engagement and that CD19 downmodulation correlates with increased *Foxo1* and *Rag1/2* expression and, thus, with functional markers of receptor editing.

### Internalized CD19 and BCR are both degraded by the lysosome and not the proteasome

**FIGURE 3.**
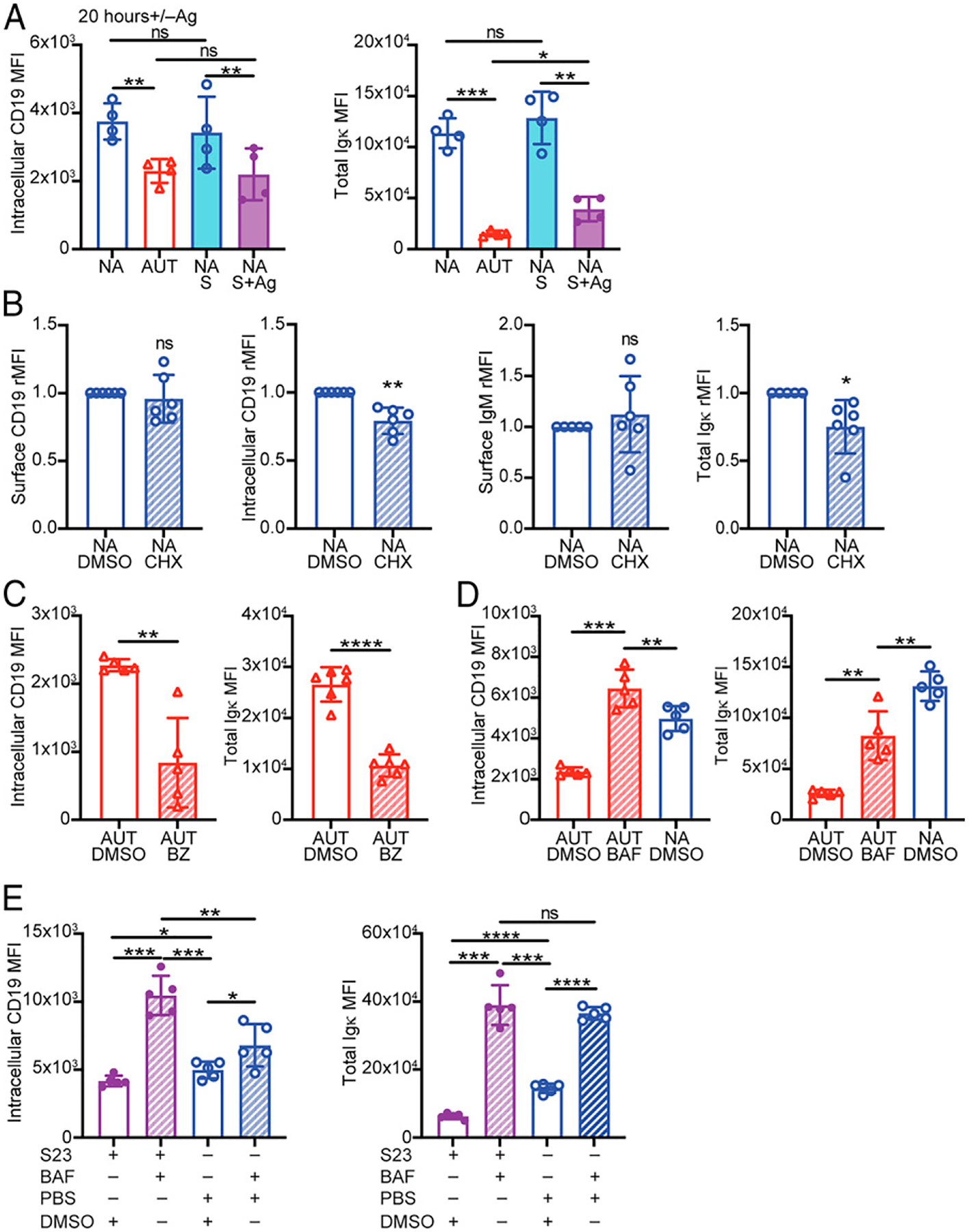
Surface CD19 molecules that internalize following BCR stimulation are degraded by the lysosome and not the proteasome. (**A**) Mean ± SD of intracellular CD19 and total Igκ MFIs (representative flow cytometry is shown in Supplemental Fig. 2B) in 3–83Igi nonautoreactive (NA) B220^+^CD24^high^CD23^−^immature B cells cultured for 20 h on K^b^ or K^d^ stromal cell layers and in ex vivo autoreactive (AUT) and NA immature B cells. Intracellular CD19 was stained separately from the surface pool by using the same anti-CD19 mAb in different colors. *n* = 4 mice/group analyzed in four independent experiments. The *p* values were calculated using a paired *t* test for all comparisons, except when compared with AUT ex vivo, in which an unpaired *t* test was used. (**B**) Mean ± SD of surface and intracellular CD19 and of surface IgM and total (surface plus intracellular) Igκ (analyzed as shown in Supplemental Fig. 2B) in B220^+^CD24^high^CD23^−^ NA immature B cells cultured for 24 h with 2 μM of the translation inhibitor cycloheximide (CHX) or DMSO control. Relative MFI (rMFI) data are from *n* = 6 mice analyzed in four independent experiments and are expressed for each sample as fold change over cells treated with DMSO. The *p* values were calculated using a one-sample *t* test. Mean ± SD of intracellular CD19 and total Igκ MFIs in 3–83Igi,H-2^b^ AUT B220^+^CD24^high^CD23^−^ immature B cells cultured for 24 h with 0.5 μM of the proteasome inhibitor bortezomib (BZ) (**C**), 12 nM of the lysosome inhibitor bafilomycin (BAF) (**D**), or DMSO control (C and D). NA cells cultured with DMSO are included in (D). *n* = 5 mice/group from five independent experiments. The *p* values were calculated using a paired *t* test, except when AUT cells were compared with NA cells, in which case an unpaired *t* test was used. (**E**) Mean ± SD of intracellular CD19 and total Igκ MFIs in NA B220^+^ CD24^high^CD23^−^ immature B cells cultured first with or without 10 μg/ml S23 Ab for 4 h and then also with 12 nM bafilomycin or DMSO control for an additional 20 h. *n* = 5 mice from five independent experiments. The *p* values were calculated using a paired *t* test. In all bar graphs, *p ≤ 0.05, **p ≤ 0.01, ***p ≤ 0.001, ****p ≤ 0.0001.

By using the same anti-CD19 Ab clone in two different colors, we performed a flow cytometric measurement differentiating between surface and intracellular pools of CD19 (Supplemental Fig. 2B). In addition, we measured total (intracellular plus surface) Igκ L chain levels as an approximate measure of intracellular BCR (Supplemental Fig. 2B). Autoreactive immature B cells showed significantly reduced levels of intracellular CD19 and of total Igκ relative to nonautoreactive cells (Fig. 3A). This result was recapitulated by culturing 3–83,H-2^d^ bone marrow B cells on K^b^ stromal cells (Fig. 3A, Supplemental Fig. 2C), suggesting that BCR stimulation of immature B cells leads to either reduced translation or increased degradation of CD19. Treatment of nonautoreactive immature B cells with the translation inhibitor cycloheximide slightly decreased intracellular CD19 and total Igκ but did not lead to a reduction of surface CD19 and IgM (Fig. 3B), indicating surface CD19 is stable for at least 24 h without de novo protein synthesis.

There are two major mechanisms by which proteins are degraded in the cell: through the proteasome system or in the lysosome (54). Treating 3–83Igi,H-2^b^ bone marrow B cells with the proteasome inhibitor bortezomib (55) for 20 h did not increase intracellular CD19 levels. Instead, bortezomib decreased both intracellular CD19 and total Igκ (Fig. 3C), likely by promoting the unfolded protein response and autophagy (56). This dose of bortezomib was nevertheless effective in increasing FOXO1 (Supplemental Fig. 2D), a protein known to be degraded by the proteasome (57).

We next investigated if CD19 is degraded by the lysosome by treating bone marrow B cells from 3–83Igi,H-2^b^ mice with the lysosome inhibitor bafilomycin (58, 59). Blocking lysosomal degradation for 20 h led to a significant increase of intracellular CD19 in autoreactive B cells to a level that was even slightly higher than that in nonautoreactive cells (Fig. 3D). Total Igκ was also significantly elevated by bafilomycin (Fig. 3D). These findings were recapitulated with 3–83Igi,H-2^d^ bone marrow B cells stimulated with the agonistic S23 Ab in the presence or absence of bafilomycin (Fig. 3E). Bafilomycin also increased intracellular CD19 and Igκ in nonstimulated (i.e., naive) 3–83 B cells (Fig. 3E).

Overall, these data demonstrate that following BCR stimulation of immature B cells, CD19 and BCR are both internalized and degraded by the lysosome and not the proteasome.

### Blocking CD19 degradation only partially restores surface CD19 and BCR expression

We next explored the possibility that lysosome-mediated CD19 degradation prevents CD19 surface reexpression. To test this, we again treated autoreactive bone marrow B cells with bafilomycin for 20 h and analyzed the levels of surface CD19.

**FIGURE 4.**
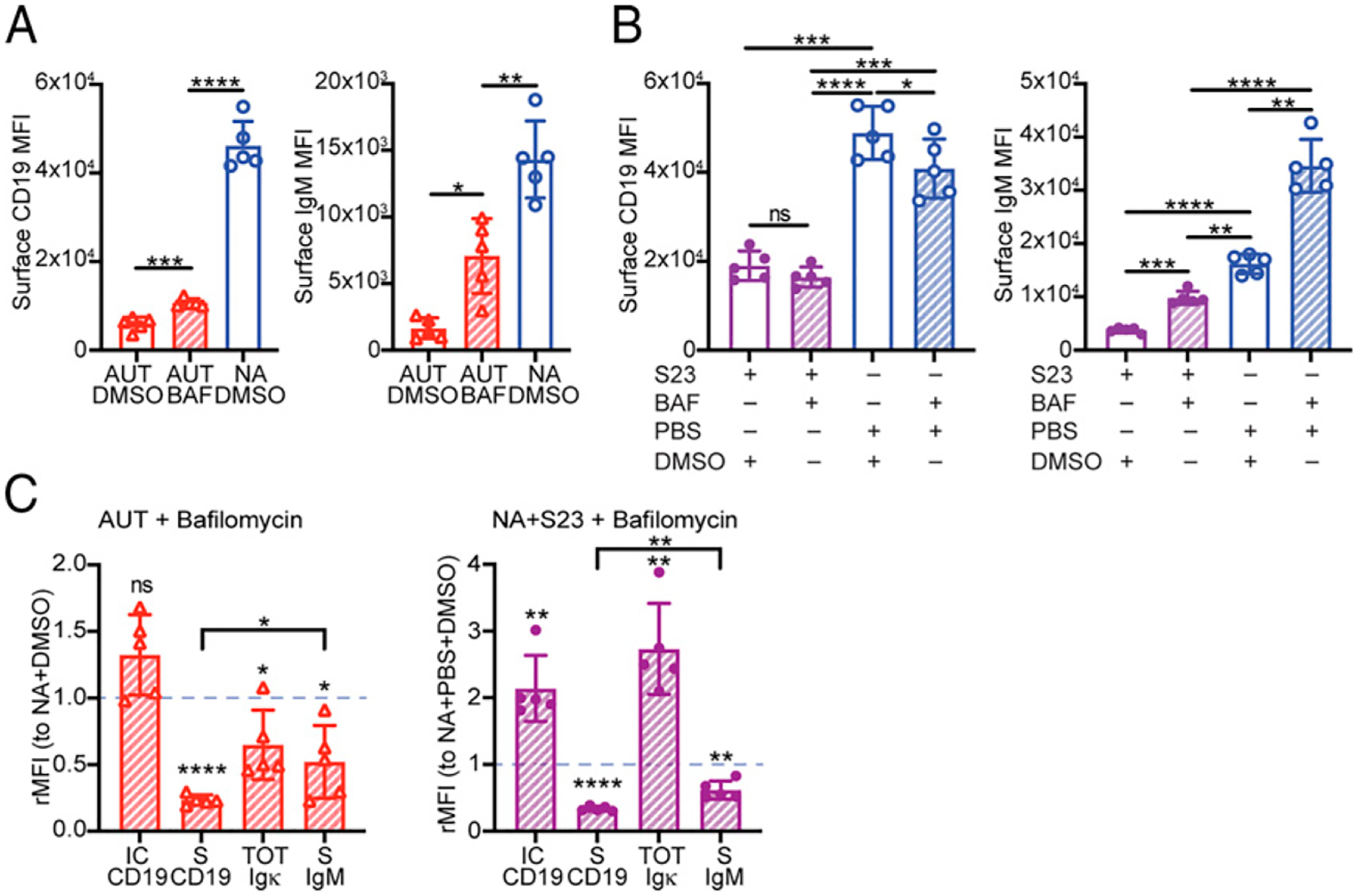
Blocking lysosomal degradation does not fully restore surface CD19 in BCR-stimulated immature B cells. (**A**) Mean 6 SD of surface CD19 and IgM on 3–83Igi,H-2^b^ autoreactive (AUT) B220^+^CD24^high^CD23^−^ immature B cells cultured for 24 h with 12 nM bafilomycin (BAF) or DMSO control and in nonautoreactive (NA) immature B cells cultured with DMSO. *n* = 5 mice analyzed in five independent experiments. The *p* values were calculated using a paired *t* test, except when compared with NA plus DMSO cells, in which case an unpaired *t* test was used. (**B**) Mean ± SD of surface CD19 and IgM in B220^+^CD24^high^CD23^−^ NA immature B cells cultured first with or without 10 μg/ml S23 Ab for 4 h and then also with 12 nM bafilomycin or DMSO control for an additional 20 h. *n* = 5 mice analyzed in five independent experiments. The *p* values were calculated using a paired *t* test. (**C**) Relative mean ± SD of intracellular CD19, surface CD19, total (surface plus intracellular) Igκ, and surface IgM relative MFI (rMFI) (analyzed as shown in Supplemental Fig. 2B) measured in AUT (left) and NA (right) B220^+^CD24^high^CD23^−^ immature B cells in culture and treated as indicated in (A) and (B). Data from *n* = 5 mice/group analyzed in five independent experiments are expressed as fold change over NA cells treated with DMSO (represented with a dashed blue line) in each individual experiment. The *p* values for differences between each experimental AUT or NA group and control NA plus DMSO samples were calculated using a one-sample *t* test and are displayed above each bar. The *p* values for differences between two AUT or two NA experimental groups were calculated using a paired *t* test and are displayed on top of lines. In all bar graphs, *p ≤ 0.05, **p ≤ 0.01, ***p ≤ 0.001, **** *p* ≤ 0.0001.

Despite the restoration of the intracellular CD19 pool (Fig. 3D), bafilomycin only slightly increased surface CD19 in autoreactive immature B cells (Fig. 4A). Bafilomycin also failed to recover any amount of surface CD19 in S23-stimulated 3–83 B cells (Fig. 4B). Interestingly, when comparing relative surface expression, recovery of surface IgM by bafilomycin was significantly better than that of CD19 (Fig. 4C). Thus, intracellular CD19 degradation only partly accounts for the low surface CD19 levels displayed by autoreactive immature B cells, suggesting the presence of additional factors involved in regulating surface CD19 expression.

### BCR stimulation and downmodulation enforces CD19 internalization and prevents CD19 surface reexpression

**FIGURE 5.**
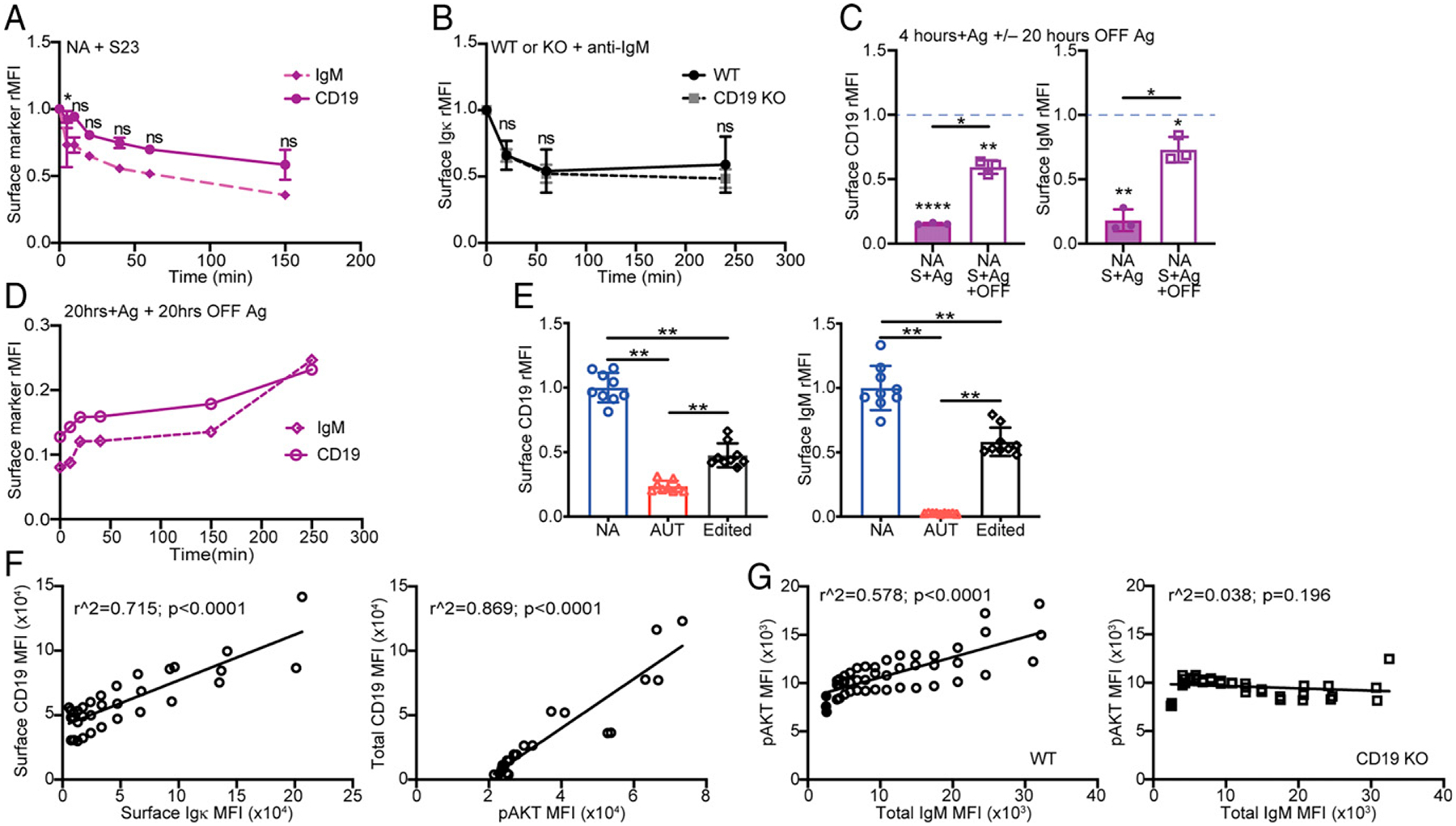
Expression of surface CD19 correlates with IgM and p-AKT. (**A**) Kinetics of surface IgM and CD19 on 3–83Igi,H-2^d^ nonautoreactive (NA) B220^+^CD24^high^CD23^−^ immature B cells that were treated with 10 μg/ml of S23 Ab for indicated times. (**B**) Kinetics of surface Igκ downregulation in immature B cells from BL/6 wild-type (WT) and CD19 knockout (KO) mice. Surface Igκ was measured on B220^+^CD24^high^CD23^−^IgD^−^ immature B cells cultured for indicated times with 5 μg/ml of anti-IgM F(ab’)_2_ antibodies. Data in (A) and (B) are mean ± SD from *n* = 3 mice/group in three independent experiments and are expressed as fold change MFI of cells treated with stimulating Ab over cells treated with PBS for each mouse at the same time point. The *p* values indicate differences in the kinetics of downregulation of IgM and CD19 in (A) and of Igκ between the two strains in (B) and were calculated using an unpaired *t* test that compared the proportion of downregulation between the two groups during each time segment. (**C**) Bone marrow B cells from NA (H-2^d^) mice (*n* = 3 mice in three experiments) were cultured for 4 h on K^b^ stromal cell layers (NA+S+Ag) and then without stromal for an additional 20 h of culture (NA+S+Ag+OFF). Bar graphs display surface CD19 and IgM MFIs (mean ± SD) from B220^+^CD24^high^CD23^−^ immature B cells cultured on K^b^ relative to control cells cultured without Ag (i.e., on K^d^ stromal), the latter being represented by a blue dashed line. Differences between cells cultured with or without Ag were calculated using a one-sample *t* test and are displayed with *p* values placed above each bar. Differences between groups, calculated with a paired *t* test, are displayed with *p* values above horizontal lines. (**D**) Kinetics of surface IgM and CD19 reexpression. Relative surface CD19 and IgM on B220^+^CD24^high^CD23^−^ NA immature B cells that were cultured on K^b^ stromal for 20 h (time 0 on graph) and then without stromal for indicated times. MFIs were normalized to NA cells cultured without Ag (i.e., on K^d^ stromal) for 20 h and then without stromal for each time point. Data are from one experiment with one mouse and are representative of three independent experiments. (**E**) Relative surface CD19 and IgM on ex vivo B220^+^CD24^high^CD23^−^ immature B cells from 3–83Igi mice that were nonautoreactive (NA; IgM^+^ cells from H-2^d^ mice), autoreactive (AUT; IgM^−/low^ cells from H-2^b^ mice), and edited (IgM^+^ cells from H-2^b^ mice). Data (mean ± SD from *n* = 9 mice analyzed in three experiments) are relative to the average values measured in NA cells. The *p* values were calculated using an unpaired Mann–Whitney *U* test. (**F**) Simple linear regression analyses between surface CD19 and Igκ (left) and total (surface plus intracellular) CD19 and intracellular p-AKT (right) in ex vivo B220^+^CD24^high^CD23^−^IgD^−^Igκ^+^ immature B cells from BL/6 mice. Small serial gates (Supplemental Fig. 3D) were made based on either surface Igκ to correlate MFIs of surface CD19 and Igκ or total CD19 to compare MFIs of total CD19 and p-AKT. Each symbol represents gated cells from each individual mouse. Data on the left are from three mice analyzed in three independent experiments. Data on the right are from two mice analyzed in one experiment; analysis of an additional mouse (*r*^2^ = 0.948; *p* < 0.0001) is not in the graph because it was obtained with an older anti–p-AKT Ab with generally lower MFIs. (**G**) Simple linear regression analysis between p-AKT and total (surface plus intracellular) IgM in bone marrow B220^+^CD24^high^CD23^−^IgD^−^B cells from indicated mice (*n* = 3 mice/group analyzed in one experiment). Small serial gates (Supplemental Fig. 3E) were made based on increasing IgM levels. Each symbol represents gated cells from each individual mouse. Filled symbols represent IgM^−^ cells. In all bar or line graphs, *p ≤ 0.05, **p ≤ 0.01.

To better understand the relationship between IgM and CD19 downregulation, we examined the kinetics of CD19 and IgM comodulation following BCR stimulation. Bone marrow 3–83 B cells from 3–83Igi,H-2^d^ mice were cultured in media with 10 μg/ml S23 or PBS and analyzed over time. At each time point, the mean fluorescence intensity (MFI) of CD19 and IgM measured on S23-stimulated immature B cells was divided by that obtained from nonstimulated B cells, and these values were plotted against time (Fig. 5A). Interestingly, the rate of CD19 and IgM internalization was almost identical in immature B cells (Fig. 5A). This was not the case in mature B cells, which internalized IgM much more rapidly than CD19 (Supplemental Fig. 3A). Nevertheless, by 4 h of stimulation, CD19 and IgM were downmodulated to similar extent in immature and mature B cells (Supplemental Fig. 3B). Of note, there was no difference in the rate of Igκ (i.e., IgM) downregulation between wild-type and CD19 knockout immature B cells (Fig. 5B), indicating that CD19 does not contribute to, and is not necessary for, BCR downregulation in these cells.

We next tested the hypothesis that continual BCR stimulation is what prevents intracellular CD19 from being expressed on the cell surface of autoreactive immature B cells. To test this idea, we cultured bone marrow B cells from 3–83Igi,H-2^d^ mice on K^b^ stromal cells for 4 h, then removed the B cells from the stromal cell layer, and transferred them into a new well with media for 20 h without additional stimulation. Surface expression of both IgM and CD19 was significantly increased 20 h after removal of the K^b^ Ag (Fig. 5C). In addition, the recovery of surface CD19 and IgM followed similar kinetics (Fig. 5D), with a rapid expression of low amounts of both proteins during the first hour of Ag removal, followed by slower reexpression kinetics that took >4 h to complete (Fig. 5C, 5D).

We then examined whether the relationship between surface BCR and CD19 also applies to immature B cells in vivo by using different mouse models. We first analyzed surface CD19 and IgM in edited, surface IgM^+^, immature B cells (gated as previously described in Ref. 60) from 3–83Igi,H-2^b^ mice. Edited B cells, which are able to leave the bone marrow expressing diverse BCRs (3-83H paired with diverse L chains) (12), displayed intermediate levels of surface IgM and CD19 relative to 3–83 autoreactive and nonautoreactive B cells (Fig. 5E). Furthermore, immature B cells from the classical B cell anergy mouse models MD4 × ML5 and Ars/A1, which are known to bind Ag with low avidity and bypass central tolerance (47, 48), showed only slightly decreased amounts of surface IgM and surface CD19 relative to nonautoreactive MD4 B cells (Supplemental Fig. 3C). Finally, we analyzed the correlation between surface CD19 and Igκ on bone marrow immature B cells from wild-type mice. Wild-type immature B cells are typically distributed along a wide range of BCR surface levels, with self-reactive clones being more prevalent among IgM-low cells (61). Even in wild-type immature B cells, the surface levels of BCR and CD19 were positively correlated (Fig. 5F, left, Supplemental Fig. 3D, left). Moreover, the degree of CD19 expression in wild-type immature B cells correlated with the intracellular total level of p-AKT (Fig. 5F, right, Supplemental Fig. 3D, right).

To establish whether the p-AKT pool that correlates with surface BCR is regulated by CD19-dependent mechanisms, we analyzed p-AKT, PTEN, and IgM levels in wild-type and CD19-deficient immature B cells. As shown in Fig. 5G, a basal level of p-AKT was present in IgM-negative developing B cells. This p-AKT level increased in IgM^+^ immature B cells with increasing amounts of IgM (Supplemental Fig. 3E) and only if the cells expressed CD19 (Fig. 5G). Furthermore, levels of the phosphatase PTEN did not proportionally decrease with increasing IgM levels (Supplemental Fig. 3F, left), indicating that the p-AKT pool that increases with IgM is not regulated by PTEN. Instead, there was a visible increase of PTEN with increasing levels of IgM in wild-type immature B cells but not in CD19-deficient cells (Supplemental Fig. 3F).

In concert, these data show that the level of surface CD19 on each bone marrow immature B cell depends on the amount of surface BCR and that, together, CD19 and IgM regulate the proportional increase of PI3K-AKT activation.

### Surface CD19 levels are modulated by PI3K signaling

**FIGURE 6.**
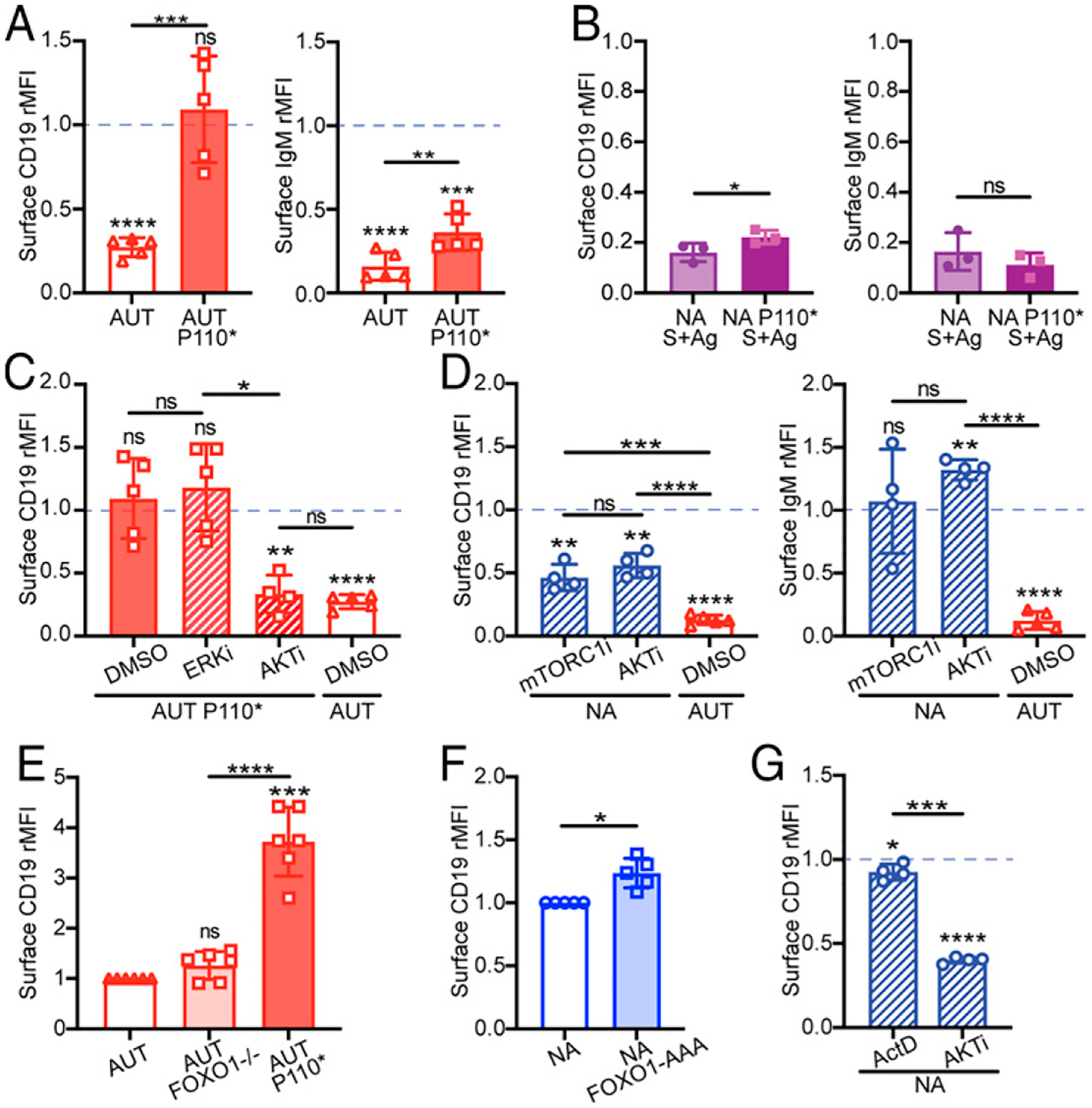
PI3K signaling positively modulates surface CD19 in immature B cells via the AKT-mTORC1 axis. (**A**) Relative surface CD19 and IgM levels on ex vivo B220^+^CD24^high^CD23^−^ immature B cells from 3–83Igi,H-2^b^ autoreactive (AUT) mice that did or did not express the active PI3Kα form P110*. Data (mean ± SD; *n* = 5 mice analyzed in four independent experiments) are expressed as fold change over ex vivo immature B cells from 3–83Igi,H-2^d^ nonautoreactive (NA) mice, represented by a blue dashed line. The *p* values were calculated using one-sample *t* test and are displayed above each bar, whereas differences between bars were calculated using an unpaired *t* test displayed on top of horizontal lines. (**B**) Relative surface CD19 and IgM measured on NA and NA-P110* B220^+^CD24^high^CD23^−^ immature B cells that were cultured for 20 h on a K^b^ stromal cell layer. Data (mean ± SD; *n* = 3 mice analyzed in three independent experiments) are expressed as fold change over NA immature B cells cultured on K^d^ stromal. The *p* values were calculated using an unpaired *t* test. (**C** and **D**) Relative surface CD19 and IgM on B220^+^CD24^high^CD23^−^ immature B cells from indicated mice that were cultured for 20 h with inhibitors for ERK (FR180204, 30 μM), AKT (afuresertib, 5 μM), or mTORC1 (rapamycin, 10 μM) and relative to cells treated with DMSO control. Data [mean ± SD; 5 mice/group from five experiments in (C) and 4 mice/group from two experiments in (D)] are expressed as fold change over NA immature B cells cultured with DMSO. The *p* values denoting differences from NA plus DMSO samples were calculated using a one-sample *t* test and are placed above each bar. Differences between bars were calculated with a *t* test and are displayed above horizontal lines. Relative surface CD19 on ex vivo B220^+^CD24^high^CD23^−^ immature B cells from 3–83Igi,H-2^b^ AUT mice (**E**) in which B cells express or not the active PI3Kα P110* or carry the deletion of FOXO1 or from 3–83Igi,H-2^d^ NA mice (**F**) in which B cells express or not the constitutively active FOXO1-AAA. Data [mean ± SD; 6 mice/group from five experiments in (E) and 5 mice/group from three experiments in (F)] are expressed as fold change over AUT immature B cells in (E) or fold change over NA immature B cells in (F). The *p* values were calculated using a one-sample *t* test and displayed above each bar. Differences between bars in (E) were calculated with an unpaired *t* test. (**G**) Relative surface CD19 on NA B220^+^CD24^high^CD23^−^ immature B cells cultured for 20 h with inhibitors for transcription (actinomycin D [ActD], 0.1 μM) or AKT (afuresertib, 5 μM). Data (mean ± SD; *n* = 4 mice/group from two experiments) are expressed as fold change over NA treated with DMSO. The *p* values were calculated using a one-sample *t* test and displayed above each graph or a paired *t* test displayed above a horizontal line. In all bar graphs, *p ≤ 0.05, **p ≤ 0.01, ***p ≤ 0.001, ****p ≤ 0.0001.

Although it is well established that CD19 coordinates with the BCR to activate PI3K-AKT in B cells (22–25), whether PI3K activity can feed back to influence CD19 protein expression is less clear. We have previously shown that introduction of a constitutively active form of PI3K (P110*) in 3–83 autoreactive immature B cells results in higher surface expression of CD19 (18). When analyzed more carefully, CD19 expression in P110* autoreactive immature B cells was at the level observed on nonautoreactive 3–83 B cells, whereas the expression of surface IgM was not restored (Fig. 6A). Interestingly, both IgM and CD19 were downmodulated when bone marrow B cells from 3–83Igi,H-2^d^ P110*-mb1Cre mice were cultured on K^b^ stromal cells (Fig. 6B), indicating that constitutive PI3K activation is unable to block Ag-mediated CD19 (and BCR) internalization.

To further explore the mechanism by which PI3K regulates CD19 expression in immature B cells, we used small-molecule inhibitors in bone marrow cell cultures. Treatment with the AKT inhibitor afuresertib, but not the ERK inhibitor FR180204, decreased CD19 expression on autoreactive 3–83,H-2^b^ B cells that harbored P110* (Fig. 6C), indicating that P110* increases CD19 expression via AKT activity. Treating bone marrow cells from 3–83Igi,H-2^d^ mice with afuresertib resulted in a significant reduction of CD19, but not of IgM (Fig. 6D), B220, or CD23 (Supplemental Fig. 4A). These results were recapitulated by treating 3–83Igi,H-2^d^ bone marrow B cells with the mTORC1 inhibitor rapamycin (Fig. 6D, Supplemental Fig. 4A), indicating that the PI3K-AKT pathway promotes and maintains CD19 surface expression in nonautoreactive immature B cells via mTORC1 activity. Importantly, the inability of the inhibitors to decrease IgM, B220, and CD23 suggests that the changes in CD19 were not due to diminished cell size or metabolic stress. These inhibitors also failed to decrease intracellular CD19 (Supplemental Fig. 4B), suggesting that their effect on surface CD19 was not the result of increased CD19 degradation or decreased CD19 translation.

Studies in pre-B cells have uncovered a transcription factor circuit modulated by PI3Kd that controls *Cd19* gene expression (62). By mediating the inhibition of FOXO1, this PI3K-AKT pathway leads to higher Pax5 expression and, consequently, higher *Cd19* transcription. To determine whether surface CD19 levels in 3–83 immature B cells are regulated via this transcriptional pathway, we measured CD19 levels on 3–83,H-2^b^ autoreactive immature B cells harboring a deletion of the *Foxo1* gene (45) and on 3–83,H-2^d^ nonautoreactive immature B cells that were either treated with the transcription inhibitor actinomycin D or that expressed the active (degradation-resistant) form FOXO1-AAA (46). In contrast to that observed with the expression of P110*, deletion of FOXO1 did not increase surface expression of CD19 in autoreactive B cells (Fig. 6E). Conversely, neither the expression of FOXO1-AAA (Fig. 6F) nor the treatment with actinomycin D (Fig. 6G) decreased surface CD19 levels in nonautoreactive B cells, even though both actinomycin D and the AKT inhibitor afuresertib caused a significant reduction of *Cd19* mRNA (Supplemental Fig. 4C). Surprisingly, expression of CD19 on mature B cells was more dependent on gene transcription than on PI3K signaling (Supplemental Fig. 4D).

In sum, these data indicate that the PI3K-AKT pathway positively modulates surface CD19 on immature B cells via mTORC1 and independently of FOXO1 and changes in *Cd19* gene transcription.

## DISCUSSION

This study was performed to investigate and account for previous observations showing that mouse and human immature B cells that express BCRs with high avidity for self-antigen and undergo central tolerance express much lower levels of surface CD19 when compared with immature B cells that do not bind self-antigen. Results from our study demonstrate that in mouse immature B cells, CD19 is downmodulated from the cell surface together with the BCR in proportion to the extent of BCR stimulation, and the remaining surface BCR coordinates with CD19 to increase the activity of the PI3K-AKT pathway. The internalized BCR and CD19 are both degraded by the lysosome, but blocking this degradation only minimally increases surface CD19. Instead, surface CD19 is positively modulated by PI3K-AKT via the mTORC1 pathway, and its restoration on immature B cells after Ag encounter occurs either by removing the autoantigen (or the self-reactive BCR via receptor editing) or enforcing activation of PI3K.

The existence of a CD19 domain that directs its association with IgM as well as the fact that CD19 on the plasma membrane moves into very close proximity with IgM following BCR stimulation have been described (28, 29, 63–66). Nevertheless, reports on CD19 comodulation with IgM have been scant. It has been previously shown that in human mature B cell lines, CD19 is downmodulated and internalized with the BCR (IgM or IgG) following BCR stimulation and that this occurs at a degree proportional to the amount of stimulation (63). Our observations in mouse primary immature B cells are remarkably similar to those findings. Intriguingly, however, the observations reported with human B cell lines were not replicated in more recent studies with primary human blood B cells (66, 67). This is surprising and may have been due to the timing of CD19 measurement in these more recent studies. In a humanized mouse model, we have observed that when human immature B cells bind a membrane self-antigen, CD19 is downmodulated similar to mouse immature B cells (41), indicating no differences between mouse and human B cells. Furthermore, mouse primary mature B cells that circulate through the bone marrow are also capable of downregulating CD19 following BCR stimulation (Supplemental Fig. 3A, 3B). Thus, our data indicate that CD19 is downregulated with the BCR in both immature and mature B cells. Interestingly, the kinetics of CD19 downmodulation in these cells were remarkably similar. In contrast, the kinetics of IgM downmodulation were quicker in mature than immature B cells, possibly because of the presence of IgD and other surface molecules that are lacking in immature B cells.

We show that after modulation and internalization, CD19 is degraded together with IgM by the lysosome, and not the proteasome. Hence, this is similar to what has been described in mature B cells, which traffic the internalized receptor via the endosome to the lysosome for Ag digestion and MHC loading and presentation (68, 69). Although in Ag-stimulated mature B cells, lysosome trafficking of the BCR is needed for presentation of some type of Ags (68), we suggest that autoreactive immature B cells internalize and degrade IgM and CD19 to reduce PI3K activation and, thus, to undergo receptor editing (18–21). Indeed, deletion of either IgM or CD19 in immature B cells results in a similar phenotype (8, 11, 36–39), which is arrest of cell differentiation and initiation of receptor editing. Our data show that wild-type immature B cells exhibit a clear proportionality between surface BCR, CD19, and p-AKT, which suggests that in these cells, the BCR proportionally modulates PI3K activity via CD19. The activity of the PI3K pathway can also be negatively modulated via the phosphatases PTEN and SHIP (reviewed in Ref. 70). We show that the expression of PTEN did not inversely correlate with IgM and p-AKT in wild-type immature B cells, suggesting that this phosphatase does not contribute to the fine modulation of the PI3K-AKT pathway that is observed in IgM^+^ bone marrow immature B cells. This finding is surprising, given that PTEN expression has been shown to inversely correlate with surface IgM in peripheral mouse and human B cells (reviewed in Ref. 71). We did not analyze the expression of SHIP and, thus, cannot exclude a role of this phosphatase in this process. Downstream of the BCR, PI3K can be activated via both CD19-dependent and -independent pathways (22–26). We show that in the absence of CD19, immature B cells fail to increase p-AKT in proportion to IgM. This clearly indicates that in immature B cells, tonic BCR signaling activates PI3K-AKT in a proportional manner via the CD19-dependent pathway. Overall, these data suggest that only immature B cells with sufficient amounts of surface BCR and CD19 are able to increase the pool of active PI3K and AKT to the degree necessary for further differentiation and selection into the periphery. They also suggest that downmodulation of CD19 in autoreactive B cells is needed to prevent PI3K activation and unwarranted escape from tolerance, although 3–83Igi autoreactive immature B cells also upregulate PTEN (5), which further decreases the activity of the PI3K pathway.

We were surprised to find that inhibition of lysosome-mediated protein degradation only minimally increased surface CD19 in autoreactive B cells, indicating the existence of dominant mechanisms that prevent surface CD19 expression. We demonstrate that the major mechanism preventing CD19 from reaching the cell surface in autoreactive cells is the chronic binding of the Ag receptor to cognate Ag. Indeed, removal of the BCR-specific Ag increased both IgM and CD19 within 20 min. The kinetics of surface reexpression of receptor and coreceptor were similar, but restoring maximal surface levels required several hours. The reasons for these long kinetics are currently unclear. Furthermore, the restoration of surface CD19 appeared to be sensitive to the presence of self-antigen, suggesting that any BCR and CD19 recycling back to the cell surface are rapidly internalized upon Ag binding. Indeed, immature B cells belonging to a polyclonal population (such as wild-type cells or 3–83Igi cells after receptor editing), and thus including clones expressing BCR with some level of self-reactivity, displayed, on average, lower levels of surface IgM and CD19 than de facto nonautoreactive immature B cells. Moreover, immature B cells from transgenic mouse models of anergy that bind self-antigen with ultralow avidity and still retain largely intact levels of surface IgM did not exhibit significant degrees of CD19 downmodulation. Therefore, our data indicate that in immature B cells, CD19 and IgM internalize together in proportion to BCR stimulation and are not significantly reexpressed until the stimulus has been removed.

As discussed above, it is well accepted that CD19 promotes activation of PI3K in B cells (22–25). In this study, we show that the activity of PI3K feeds back to positively regulate surface expression of CD19, thus creating a self-reinforcing signaling loop. Indeed, inhibiting either AKT or mTORC1 in nonautoreactive cells reduces their surface CD19 expression by half, whereas activating PI3K (via expressing P110*) in autoreactive cells increases CD19 expression to levels similar of those in nonautoreactive cells, without being accompanied by an equivalent restoration of surface IgM. The contribution of the PI3K-AKT-mTORC1 pathway to CD19 surface expression appeared to be largely independent of its inhibiting role in autophagy (72). This is indicated by the finding that AKT and mTORC1 inhibitors did not alter intracellular CD19 levels and that direct inhibition of the lysosome (i.e., autophagy) did not greatly modify surface CD19 in either autoreactive or nonautoreactive cells. The PI3K-CD19 pathway that regulates surface CD19 appears to be also largely independent of its effect on gene transcription and distinct from that previously described in pre-B cells by which FOXO1 inhibits the transcription of *Cd19* (62). In immature B cells, in fact, deletion of FOXO1 did not increase surface CD19 in autoreactive cells, and expression of a degradation-resistant FOXO1 did not decrease surface CD19 in nonautoreactive cells. Moreover, although the transcription inhibitor actinomycin D and the AKT inhibitor afuresertib both drastically reduced *Cd19* mRNA levels in nonautoreactive immature B cells, only afuresertib significantly reduced surface CD19 expression. Lack of CD19 changes were also observed when nonautoreactive cells were treated with the protein translation inhibitor cycloheximide, indicating that CD19 protein is relatively stable within a 24-h period. Nevertheless, it remains challenging to reconcile that, while in vivo, the expression of P110* elevates surface CD19 in autoreactive cells to levels observed in nonautoreactive cells, in vitro, it does not prevent Ag-induced CD19 downmodulation. It might be that with sufficient time within a chronic setting (i.e., chronic BCR stimulation in bone marrow tissue), P110* is able to restore surface CD19 (but not IgM) via multiple mechanisms –increasing *Cd19* gene transcription, inhibiting lysosome-mediated protein degradation, and increasing CD19 transportation to the plasma membrane and plasma membrane formation, each contributing by a small degree to surface expression. Interestingly, the mechanisms of maintenance of CD19 on mature (recirculating) B cells appeared to be different from those of immature B cells, as surface CD19 on mature B cells was more dependent on gene transcription and less on PI3K. Furthermore, we show that PI3K and lysosome-mediated protein degradation influence surface IgM differently than CD19. Lysosome-mediated protein degradation has a significant role in preventing surface IgM expression in both autoreactive and nonautoreactive immature B cells, whereas changes in PI3K activity have very mild consequences. Although our findings make it clear that PI3K modulates surface CD19 expression in immature B cells, the mechanism by which this happens remains unresolved. One possibility is that PI3K alters the actin cytoskeleton needed to internalize CD19, transport CD19 onto the cell surface, and/or build the specific membrane corrals in which CD19 has been shown to reside (73, 74).

In summary, our data demonstrate that in immature B cells, IgM and CD19 downmodulate from the cell surface, internalize, and traffic to the lysosome with similar kinetics and in proportion to the amount of BCR bound to Ag. Receptor and coreceptor are digested by the lysosome and are not reexpressed on the plasma membrane until the Ag has been removed. Furthermore, the IgM and CD19 receptors that remain on the cell surface coordinate to induce a proportional activation of the PI3K-AKT pathway, a signaling event known to stop receptor editing and promote cell selection into the periphery. By a mechanism that requires the activation of mTORC1 but not the inhibition of FOXO1, PI3K activity feeds back into increasing CD19 surface expression, thus likely reinforcing the CD19-PI3K signaling loop that is needed for bypassing central tolerance and being selected into the periphery.

## Supplementary Material

1
