# Supplementary material for: CD19 Is Internalized Together with IgM in Proportion to B Cell Receptor Stimulation and Is Modulated by Phosphatidylinositol 3-Kinase in Bone Marrow Immature B Cells
Source: Immunohorizons. Author manuscript; Available in PMC 2023 Apr 5. (PMC10074640; doi:10.4049/immunohorizons.2200092)
Supplement: Supplementary file 1 [file NIHMS1887618-supplement-1.pdf]

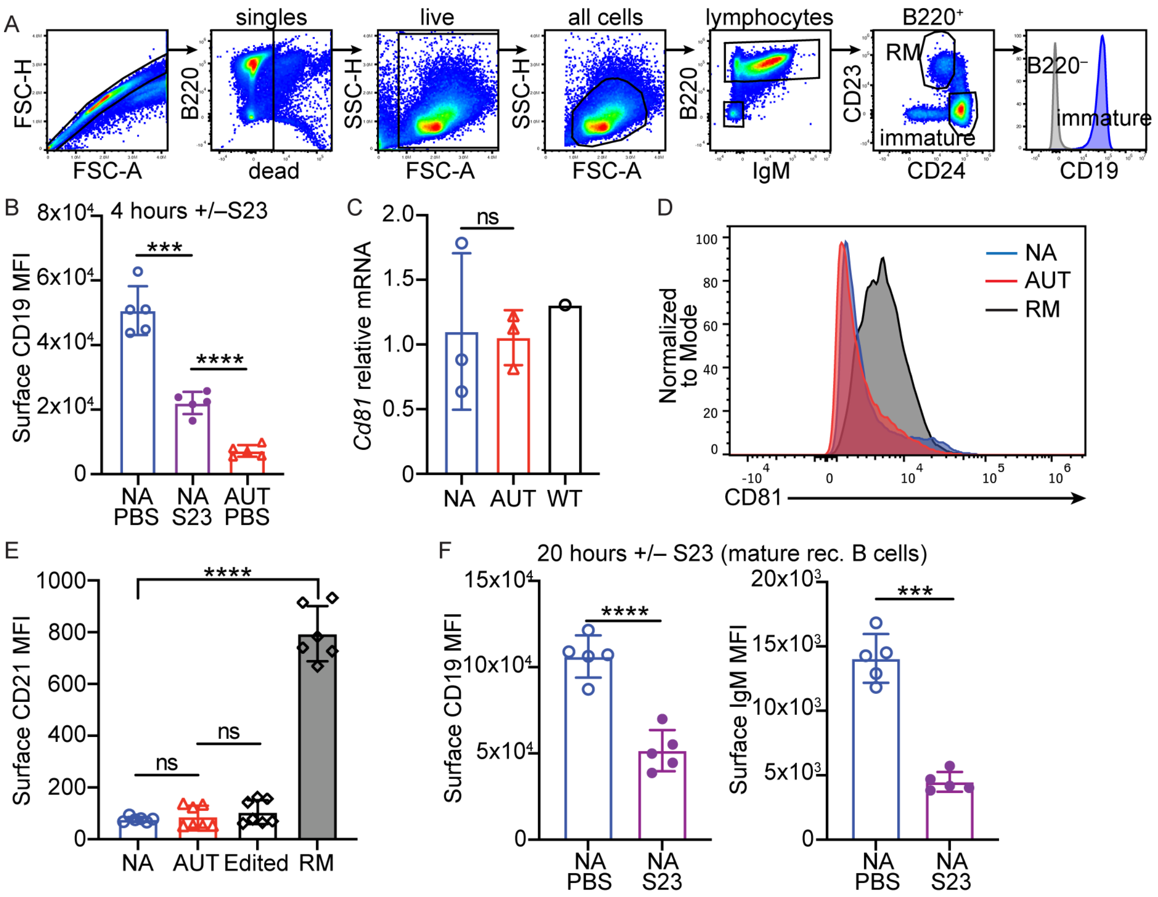

Supplementary Figure 1: CD19 is downmodulated by BCR stimulation in both immature and mature B cells. A) Representative serial gating of bone marrow cells (from 3-83Igi,H-2^d mice) for the analysis of immature and recirculating mature (RM) B cells and of CD19. This sample was magnetically pre-enriched for $B220^+$ cells. B) Levels of surface CD19 on 3-83Igi,H-2^d nonautoreactive (NA) or 3-83Igi,H-2^b autoreactive (AUT) $B220^+CD24^{high}CD23^-$ immature B cells after 4 hours of culture with 10 µg/ml S23 Ab (anti-3-83Ig) or PBS. Data (mean ± SD) are from 5 mice per group in 5 experiments. P values were calculated using a one-tailed paired t test except when compared to AUT mice, in which case an unpaired t test was used. C) Relative *Cd81* mRNA levels (mean ± SD) in $B220^+IgD^-$ cells magnetically enriched from the bone marrow of NA (n=3) and AUT (n=3) 3-83Igi mice and one WT CB17 mouse. Cells were isolated in three independent experiments, but samples were run by RT-PCR at the same time to minimize technical variations. *Cd81* mRNA was normalized to *Cd79b* mRNA in each mouse, and the normalized *Cd81* levels are expressed as fold change over the average *Cd81* levels in NA cells. P values were calculated using a one-tailed Mann-Whitney U test. D) Representative flow cytometric analysis of surface CD81 on *ex-vivo* immature B cells from AUT (red) and NA (blue) mice and from RM cells (gray). E) Surface CD21 levels on *ex-vivo* 3-83Igi immature B cells that were NA (IgM^+ from H-2^d mice), AUT ($IgM^{-/low}$ from H-2^b mice) or edited cells (IgM^+ from H-2^b mice). RM B cells from H-2^d mice are shown in gray. Data (mean ± SD) are from 6-7 mice per group analyzed in at least 5 experiments. P values were calculated using a one-tailed Mann-Whitney U test. F) Surface CD19 expression measured on 3-83 NA RM B cells after 20 hrs culture with 10 µg/ml of S23 Ab (anti-3-83Ig) and relative to PBS control. Data (mean ± SD) are from 5 mice analyzed in 5 independent experiments. P values were calculated using a one-tailed paired t test. In all bar graphs, ***, P ≤ 0.001; ****, P ≤ 0.0001; ns, not significant.

Downloaded from http://journals.aai.org/immunohorizons/article-supplement/237661/pdf/immunohorizons2200092-sup-immunohorizon_2200092_supplemental_1/ by University of Colorado Health Sciences Library user on 13 January 2023

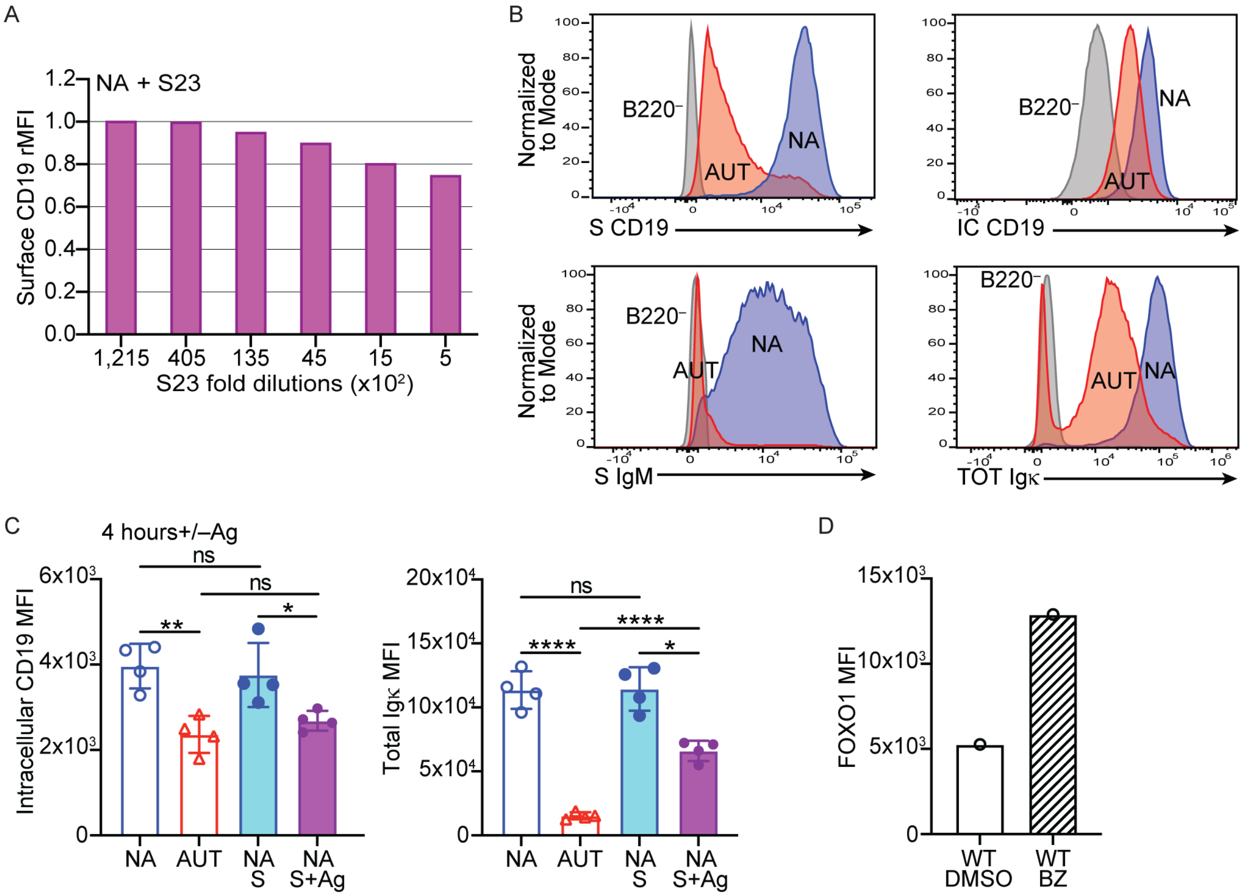

Supplementary Figure 2: CD19 downregulation by low and high avidity BCR stimulation. A) Relative surface CD19 on 3-83Igi,H-2^d nonautoreactive (NA) $B220^+CD24^{high}CD23^-$ immature B cells measured after 20 minutes incubation with 3-fold serial dilutions of S23 Ab (anti-3-83Ig) starting at 1:500 (corresponding to 10 μg/ml) and ending at 1:121,500 (corresponding to 0.082 ng/ml). Data are expressed as fold change over cells treated with PBS, from one mouse. B) Representative flow cytometric analysis of surface (S) and intracellular (IC) CD19 and of surface IgM and total (TOT) Igκ in $B220^+CD24^{high}CD23^-$ immature B cells from 3-83Igi H-2^d nonautoreactive (NA, blue) and H-2^b autoreactive (AUT, red) mice. $B220^-$ cells are in gray for comparison. Surface and intracellular CD19 pools were distinguished by fist staining surface CD19 and then intracellular CD19 with the same anti-CD19 antibody clone in different colors. C) Intracellular CD19 and total Igκ MFIs in NA $B220^+CD24^{high}CD23^-$ immature B cells after 4 hrs culture on a K^b (NA+S+Ag) or a K^d (NA+S) stromal cell layer and relative to *ex-vivo* NA and AUT cells. Data (mean ± SD) are from 4 mice per group analyzed in 4 experiments. P values were calculated using a one-tailed paired t test for all NA comparisons and a one-tailed unpaired t test when NA groups were compared to AUT cells. D) Intracellular FOXO1 MFI measured by flow cytometry in permeabilized WT (CB17) bone marrow immature B cells (n=1) cultured for 20 hrs with 0.5 μM bortezomib or DMSO control. In all bar graphs: *, P ≤ 0.05; **, P ≤ 0.01; ****, P ≤ 0.0001; ns, not significant.

Downloaded from http://journals.aai.org/immunohorizons/article-supplement/237661/pdf/immunohorizons2200092-sup-immunohorizon_2200092_supplemental_1/ by University of Colorado Health Sciences Library user on 13 January 2023

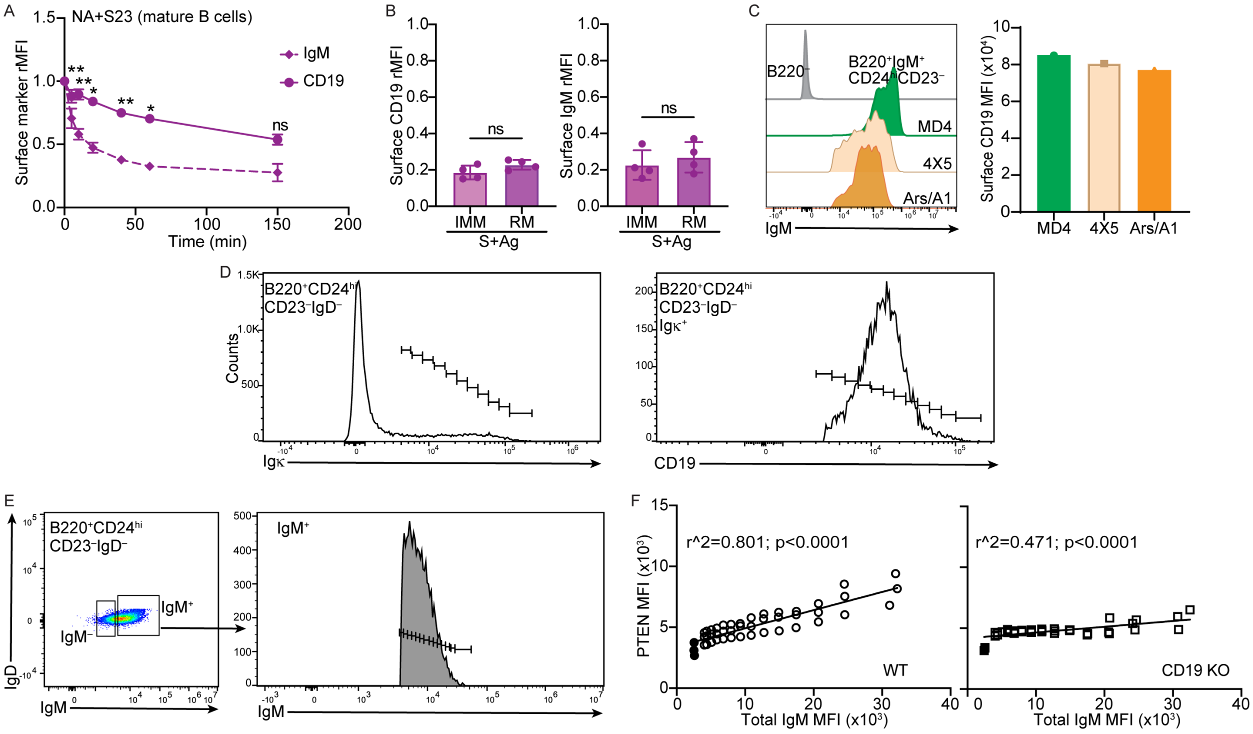

Supplementary Figure 3: CD19 and IgM downmodulation and relationship with phospho-AKT and PTEN. A) Surface IgM and CD19 measured on 3-83Igi,H-2^{d} nonautoreactive (NA) $B220^{+}CD24^{low}CD23^{+}$ mature recirculating B cells treated with 10 μg/ml of S23 Ab for indicated times. Data (mean ± SD from 3 mice in 3 experiments) are expressed as fold change MFI over NA cells treated with PBS for each time point. P values indicate differences in the kinetics of IgM and CD19 downregulation and were calculated using a one-tailed paired t test that compared the proportion of downregulation of IgM and CD19 during each time segment. B) Surface CD19 and IgM on $B220^{+}CD24^{high}CD23^{-}$ immature (IMM) and $B220^{+}CD24^{low}CD23^{+}$ mature (RM) B cells from NA mice cultured for 4 hours on K^{b} stromal cell layers (S+Ag) and relative to cells cultured on K^{d}. N = 4 mice per group in 4 experiments. C) Flow cytometric analysis of surface IgM expression (left) and surface CD19 quantification (right) on *ex-vivo* $B220^{+}CD24^{high}CD23^{-}$ IgM^{+} immature B cells from MD4, MD4xML5 (4X5), and Ars/A1 mice (n = 1 mouse per strain). $B220^{-}$ cells are shown in gray for comparison. D) Representative serial gating of surface Igκ (left) and total (surface+intracellular) CD19 (right) in immature B cells from WT BL/6 mice (gated as indicated) to measure the correlation between Igκ and CD19 or of CD19 and pAKT shown in Fig. 5F. E) Representative gating of IgM^{-} and IgM^{+} immature B cells from WT mice and serial gating based on (total) IgM expression in IgM^{+} cells. F) Simple linear regression between PTEN and IgM in bone marrow $B220^{+}CD24^{high}CD23^{-}IgD^{-}IgM^{+}$ permeabilized B cells from WT and CD19 KO mice (n = 3 mice per group analyzed in one experiment). Cells were gated on increasing total IgM levels as shown in E. Each symbol represents gated cells from each individual mouse. Filled symbols represent IgM^{-} cells as gated in E. In all bar graphs, *, P ≤ 0.05; **, P ≤ 0.01; ***, P ≤ 0.001; ns, not significant.

Downloaded from http://immunohorizons.aai.org/immunohorizons/article-supplement/237661/pdf/immunohorizons2200092-sup-immunohorizon_2200092_supplemental_1/ by University of Colorado Health Sciences Library user on 13 January 2023

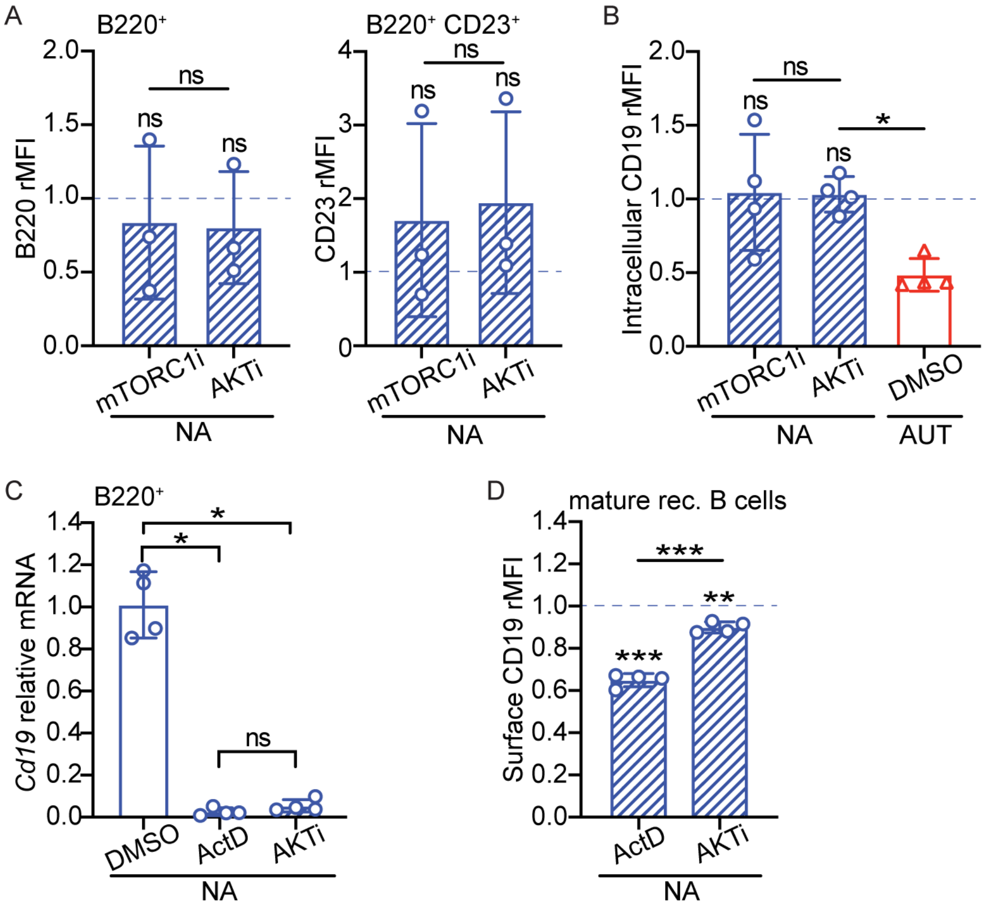

Supplementary Figure 4: Contribution of PI3K and gene transcription on the expression of CD19 and other B cell markers. A, B) Relative expression of B220 in B220$^+$ cells (A, left), of CD23 in B220$^+$CD23$^+$ cells (A, right), and of intracellular CD19 in B220$^+$CD24^{high}CD23$^-$ cells among 3-83Igi,H-2^d nonautoreactive (NA) bone marrow B cells cultured for 20 hrs with inhibitors for mTORC1 (rapamycin, 10 µM) or AKT (afuresertib, 5 µM), or 3-83Igi,H-2^b autoreactive (AUT) B cells cultured with DMSO. Data (mean ± SD, from 3 mice per group analyzed in one experiment in A, or from 4 mice per group analyzed in 2 experiments in B, are expressed as fold change over NA immature B cells cultured with DMSO (represented by a blue dashed line). P values denoting differences from NA+DMSO samples (represented by a dashed blue line) were calculated using a one-sample t test and placed above each bar. Differences between bars were calculated with a one-tailed Mann-Whitney U test and are displayed above horizontal lines. C) Relative *Cd19* mRNA levels in NA bone marrow B220$^+$ cells (n=3) cultured for 20 hours with either a transcription inhibitor (actinomycin D, 0.1 µM), an AKT inhibitor (afuresertib, 5 µM) or DMSO. Data (mean ± SD from 3 mice analyzed in two independent experiments) were normalized to *Cd79b* mRNA of cells treated with DMSO and are expressed as fold change over the average mRNA of NA cells with DMSO. P values were calculated using a one-tailed paired t test. D) Relative surface CD19 measured on 3-83Igi,H-2^d NA bone marrow recirculating mature B cells (gated as B220$^+$CD24^{low}CD23$^+$) cultured for 20 hrs with 0.1 µM actinomycin D, 5 µM afuresertib, or with DMSO. Data (mean ± SD from 4 mice analyzed over 2 independent experiments) are expressed as fold change over cells treated with DMSO. P values for differences from NA+DMSO cells (indicated by a dashed blue line) were calculated using a one-sample t test and are placed above each bar. Differences between bars were calculated with a one-tailed paired t test and are placed above the horizontal line. In all bar graphs, **, P ≤ 0.01; ***, P ≤ 0.001; ****, P ≤ 0.0001; ns, not significant.

Downloaded from http://journals.aai.org/immunohorizons/article-supplement/237661/pdf/immunohorizons2200092-sup-immunohorizon_2200092_supplemental_1/ by University of Colorado Health Sciences Library user on 13 January 2023
